# Low IgG Seroconversion among Persons Vaccinated against Measles, Republic of Congo

**DOI:** 10.3201/eid3101.240911

**Published:** 2025-01

**Authors:** Yanne Vanessa Thiécesse Mavoungou, Léa Gwladys Gangoué, Félix Koukouikila-Koussounda, Cynthia Badzi Nkoua, Pembe Issamou Mayengue, Jean-Medard Kankou, Pathou Christelle Kiminou, Princesse Mahoukou, Louis Régis Dossou-Yovo, Gabriel Ahombo, Fabien Roch Niama

**Affiliations:** Laboratoire National de Santé Publique, Brazzaville, Republic of Congo (Y.V.T. Mavoungou, L.G. Gangoué, F. Koukouikila-Koussounda, C. Badzi Nkoua, P.I. Mayengue, P.C. Kiminou, P. Mahoukou, L.R. Dossou-Yovo, F.R. Niama); Université Marien Ngouabi, Brazzaville (Y.V.T. Mavoungou, L.G. Gangoué, F. Koukouikila-Koussounda, C. Badzi Nkoua, P.I. Mayengue, G. Ahombo, F.R. Niama); Direction of Epidemiology and Disease Control, Brazzaville (J.-M. Kankou)

**Keywords:** measles, vaccines, surveillance, seroconversion, IgG, viruses, Republic of Congo

## Abstract

We report a low (38.7%; p<0.0001) level of IgG seroconversion in patients who were positive for measles virus IgM in the Republic of Congo, despite a history of vaccination. Considering this country’s recurring measles epidemics, more effective immunization strategies, including vaccine delivery methods, are needed to prevent measles outbreaks.

Under ideal conditions, the efficacy of a single dose of measles vaccine is ≈85% when administered to a 9-month-old child and 90%–95% when administered to a 12-month-old child. The World Health Organization recommends the vaccine should be given in 2 doses and at a minimum vaccination coverage of 95% for each country (*1*). However, despite high vaccine efficacy under trial conditions and the widespread use of the 2-dose schedule worldwide, vaccine effectiveness is lower and much more variable in practice (*2*). Indeed, it has been shown that responses to measles vaccines vary among persons and some vaccinated children are unable to produce the immune responses necessary for protection against measles (*3*). The immune response to measles vaccination is thought to be influenced by various host factors, including antibodies acquired through maternal antibody transfer, host-specific genetic factors, HIV infection, malnutrition, and other forms of immunosuppression (*4*). The measles vaccine failure rate is ≈10% in developed countries but can be >30% in resource-limited countries (*3*,*5*).

Numerous difficulties, such as geographic inaccessibility of certain areas, have been cited as factors favoring the persistence of measles in many countries in Africa (Republic of Congo Ministry of Health and Population, unpub. data). Those factors could have a substantial effect on the ability to eliminate measles from the continent. The Republic of Congo is part of the World Health Organization’s strategic plan to eliminate measles in Africa and has implemented a multiyear plan to fight against measles, mumps, and rubella as one of its strategic objectives. The country has introduced the combined measles/mumps/rubella vaccine, which is administered in 2 doses to children, 1 dose at 9 months and 1 dose at 15 months of age (Republic of Congo Ministry of Health and Population, unpub. data). Despite those efforts, measles continues to circulate, prompting multiple large-scale vaccination campaigns. Recent surveillance data has shown a considerable proportion of measles cases among vaccinated persons. The detection of infection among persons assumed to be vaccinated could pose a challenge to the country’s efforts to eliminate the disease. 

We hypothesized that vaccinated persons who tested positive for measles were not seroconverting. As part of routine measles surveillance activities, we determined the IgG seroconversion rates of vaccinated persons with confirmed measles. Using ELISAs, we analyzed a cohort of 191 patients who were IgM-positive for measles virus during 2020–2022 and who had a history of vaccination (>1 vaccine dose). We determined serologic differences between the last vaccination date and the date of illness onset that were >2 months apart. The significance level was set at p<0.005.

The median patient age was 4 (interquartile range 2–7) years; children <5 years of age accounted for 101 (52.9%) patients, and that age group showed a significant difference in IgG seroconversion rate (p<0.0001) (Table). For most patients, the median interval between the date of disease onset and date of last vaccination was 23 (interquartile range 4–53) months; most (n = 111 [58.1%]) patients had an interval of 2–48 months. The overall seroconversion rate was 38.7% (p<0.0001). Among patients who received 1 dose of vaccine, only 57 (38%) seroconverted (p<0.0001), and we did not observe a significant difference in seroconversion among patients who received >2 doses (p = 0.1221). Among persons who received >2 doses of vaccine, only 41.5% seroconverted compared with 58.5% who remained IgG negative (Table).

**Table:** IgG seroconversion status in study of low IgG seroconversion among persons vaccinated against measles, Republic of Congo*

Variables	IgG positive	IgG negative	p value
Total no. patients	74 (38.7)	117 (61.3)	<0.0001
Age group, y			
Median age (IQR)	5 (3–7)	4 (2–6)	0.3956
˂5	36 (35.6)	65 (64.4)	<0.0001
5–9	26 (40)	39 (60)	0.0226
>10	12 (48)	13 (52)	0.7773
Patient sex			
M	34 (39.1)	53 (60.9)	0.004
F	40 (38.5)	64 (61.5)	0.0009
Vaccination status			
1 dose	57 (38)	93 (62)	<0.0001
>2 doses	17 (41.5)	24 (58.5)	0.1221
Interval between last vaccination and disease onset, mo			
Median interval (interquartile range)	30 (5–60)	19 (3–51)	0.1957
Unknown	9 (31)	20 (69)	0.0039
2–48	42 (37.8)	69 (62.2)	0.0003
>48–108	15 (38.5)	24 (61.5)	0.0415
>108	8 (66.7)	4 (33.3)	0.1025
Municipality			
Urban	43 (33.6)	85 (66.4)	<0.0001
Rural	31 (49.2)	32 (50.8)	0.8586

*Values are no. (%) except as indicated.

We also estimated the effect of the interval between the date of disease onset and date of last vaccination on IgG production. A longer interval increased the IgG production rate, although the number of persons who produced IgG was significantly lower (p<0.0001) (Table).

Our findings revealed a high number of patients who only received 1 dose of measles vaccine, indicating a need to reinforce the booster and postvaccination follow-up system for vaccinated children. Overall, a relatively low rate of IgG seroconversion was observed in both single- and double-dose vaccine recipients. Similar results were observed in Turkey, where low IgG seroconversion against measles virus was observed in children >9 months of age (*6*). Reasons for the low number of persons who underwent IgG seroconversion after receiving >2 vaccine doses remain unclear. This finding might be linked to problems with the cold chain system of transport and storage or exposure of vaccines to light, because the measles vaccine is photosensitive; thus, inadequate training of personnel might be partially responsible for low seroconversion numbers (*7*). In large-scale vaccination campaigns, persons who do not seroconvert could be vaccine nonresponders (*8*). IgG levels have also been shown to decrease below the protective threshold in persons 10–14 years of age who received their 2 doses of vaccine at 8 and 18 months of age (*9*).

In conclusion, considering the recurring measles epidemics in the Republic of Congo, the findings from this study raise many questions about the effectiveness of the country’s measles vaccination strategy. Effective administration of vaccines and immunization strategies are needed to prevent outbreaks and might be more effective than vaccination campaigns that interrupt measles virus transmission during ongoing outbreaks.
